# Keratin Scaffolds Containing Casomorphin Stimulate Macrophage Infiltration and Accelerate Full-Thickness Cutaneous Wound Healing in Diabetic Mice

**DOI:** 10.3390/molecules26092554

**Published:** 2021-04-27

**Authors:** Marek Konop, Anna K. Laskowska, Mateusz Rybka, Ewa Kłodzińska, Dorota Sulejczak, Robert A. Schwartz, Joanna Czuwara

**Affiliations:** 1 Laboratory of Centre for Preclinical Research (CePT), Department of Experimental Physiology and Pathophysiology, Medical University of Warsaw, 02-097 Warsaw, Poland; mateuszrybka@mp.pl; 2 Mossakowski Medical Research Centre, Department of Neuropeptides, Polish Academy of Sciences, 02-106 Warsaw, Poland; 3 Department of Dermatology, Medical University of Warsaw, 02-091 Warsaw, Poland; jczuwara@yahoo.com; 4 Centre for Preclinical Research and Technology (CePT), Department of Pharmaceutical Microbiology, Faculty of Pharmacy, Medical University of Warsaw, 02-091 Warsaw, Poland; anna.laskowska@wum.edu.pl; 5 Department of Analytical Chemistry and Instrumental Analysis, Institute of Sport-National Research Institute, 03-301 Warsaw, Poland; ewa.klodzinska@insp.waw.pl; 6 Department of Experimental Pharmacology, Mossakowski Medical Research Institute, Polish Academy of Sciences, 02-106 Warsaw, Poland; dsulejczak@imdik.pan.pl; 7 Dermatology and Pathology, Rutgers New Jersey Medical School, Newark, NJ 07103, USA; raschwartz@gmail.com

**Keywords:** casomorphin, diabetes, keratin dressing, opioids, tissue regeneration, skin wound healing

## Abstract

Impaired wound healing is a major medical challenge, especially in diabetics. Over the centuries, the main goal of tissue engineering and regenerative medicine has been to invent biomaterials that accelerate the wound healing process. In this context, keratin-derived biomaterial is a promising candidate due to its biocompatibility and biodegradability. In this study, we evaluated an insoluble fraction of keratin containing casomorphin as a wound dressing in a full-thickness surgical skin wound model in mice (*n* = 20) with iatrogenically induced diabetes. Casomorphin, an opioid peptide with analgesic properties, was incorporated into keratin and shown to be slowly released from the dressing. An in vitro study showed that keratin-casomorphin dressing is biocompatible, non-toxic, and supports cell growth. In vivo experiments demonstrated that keratin-casomorphin dressing significantly (*p* < 0.05) accelerates the whole process of skin wound healing to the its final stage. Wounds covered with keratin-casomorphin dressing underwent reepithelization faster, ending up with a thicker epidermis than control wounds, as confirmed by histopathological and immunohistochemical examinations. This investigated dressing stimulated macrophages infiltration, which favors tissue remodeling and regeneration, unlike in the control wounds in which neutrophils predominated. Additionally, in dressed wounds, the number of microhemorrhages was significantly decreased (*p* < 0.05) as compared with control wounds. The dressing was naturally incorporated into regenerating tissue during the wound healing process. Applied keratin dressing favored reconstruction of more regular skin structure and assured better cosmetic outcome in terms of scar formation and appearance. Our results have shown that insoluble keratin wound dressing containing casomorphin supports skin wound healing in diabetic mice.

## 1. Introduction

Impaired wound healing is a major medical problem, especially in diabetics. Pain associated with chronic non-healing wounds can be particularly difficult to manage. Many patients experience pain despite the use of systemic analgesics. Opioids are the basic analgesic drugs employed for moderate to severe pain management [1,2]. To improve healing conditions, doctors and scientists try to create an ideal wound dressing model to accelerate healing and relieve pain symptoms. Several naturally derived materials such as collagen, cellulose, chitosan, or keratin have gained increased attention in the biomedical world [3].

In this context, keratin biomaterials play an important role in tissue engineering and regenerative medicine. Keratins are the major component of hair, wool, hooves, or feathers. Several different extraction methods allow the creation of various forms of soluble or insoluble keratin biomaterials such as films, sponges, gels, hydrogels, and scaffolds [4]. Keratin biomaterials possess unique properties such as biocompatibility, biodegradability, resistance to inflammation, and participation in skin morphogenesis and regeneration. Moreover, keratin contains the binding motif sequences RGD (Arg-Gly-Asp) and LVD (Leu-Asp-Val), which are also found in extracellular matrix proteins such as fibronectin, laminin, collagens, among others [5,6]. The heterogeneous structure of the keratin scaffolds facilitates cell adhesion and allows surface modification with analgesic or antibacterial agents [7,8].

For decades, opioids have been used as analgesic drugs for acute or chronic pain treatment to relieve suffering in patients with cancer or after surgical interventions [9]. Several studies describe the topical application of opioids, especially morphine as a strategy for pain reduction associated with skin ulcers [10], burns [11], or oral mucositis [12]. Another opioid peptide that possesses a pain relief effect is β-casomorphin. It is a biologically active peptide derived from milk β-casein and plays a vital role in improving growth performance and immunity [13,14,15]. It consists of 7 amino acids and has numerous biological activities, including reduction of fasting blood glucose, inhibition of oxidative stress, and increase of growth-related hormones. Evidence indicates that β-casomorphin can attenuate oxidative stress damage induced by hyperglycemia [16,17]. However, only a few studies have described the use of opioids as wound healing agents, and with the obtained results being inconclusive [1,18,19].

Because the direct effect of opioids on the wound healing process remains unknown, we decided to examine the result of keratin scaffolds coated with casomorphin as a wound dressing in a full-thickness skin wound model in diabetic mice.

## 2. Results

### 2.1. Monitoring of the Release of Casomorphin from Examined Dressing

In this study, the release of casomorphin from keratin dressing was measured. The capillary electrophoresis (CE) method was used to evaluate the release of examined opioids from investigated wound dressings.

On the electropherogram obtained for FKDP dressing (basic dressing), no signal originated from casomorphin was observed (Figure S1, Supplementary Materials). It was observed that casomorphin is slowly released from keratin dressing. Semi-quantitative results obtained using the CE-based method are summarized in Table 1.

### 2.2. Effect of Experimental Dressing on Viability and Migration of Murine Fibroblasts

**Figure 1:**
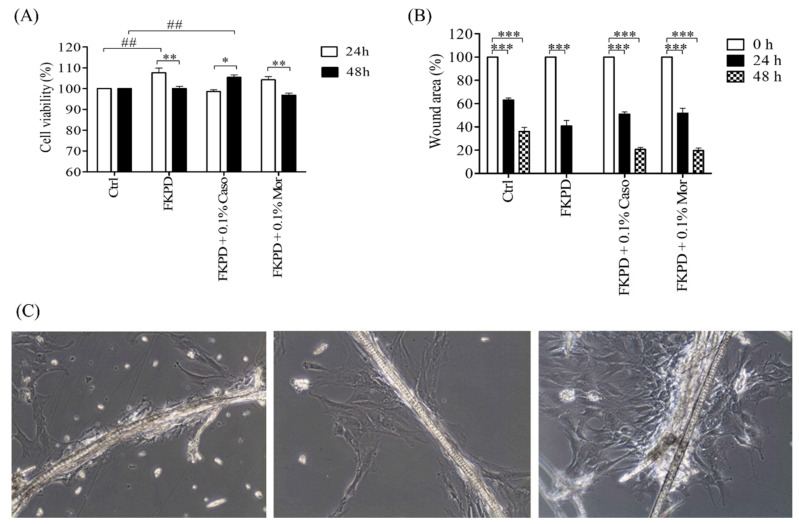
Effect of experimental dressings on cell viability (**A**) and migration (**B**) after 24 and 48 h. (**C**) NIH/3T3 cells growth on keratin biomaterials. (#-comparison control vs. treatment group) The data are statistically significant when *p* < 0.05, were: * *p*-value < 0.05, ** *p*-value < 0.01, ^##^ *p*-value < 0.01, *** *p*-value < 0.001), two-way analysis of variance, followed by Bonferroni post hoc tests, mean ± standard error of the mean).

Opioid incrusted keratin dressings were non-toxic and did not decrease cell viability. After 24 h incubation, the viability of cells treated with FKPD and FKPD + 0.1%Mor was higher than the viability of control cells. FKPD + 0.1%Caso treated cells showed viability comparable to untreated cells. After 48 h of incubation, cells treated with FKPD + 0.1%Caso showed increased viability (##) when compared to the control cells. It was observed that the viability of cells treated with FKPD and FKPD + 0.1%Mor decreased (**) after 48 h and was comparable with the viability of the control cells (Figure 1A).

Cells treated with experimental keratin dressings migrated faster than control cells. The highest rate of migration was observed for cells treated with FKPD. There was no significant difference in migration rate for cells treated with FKPD-0.1% Caso and FKPD-0.1% Mor (Figure 1B).

### 2.3. Diabetes Studies

**Figure 2:**
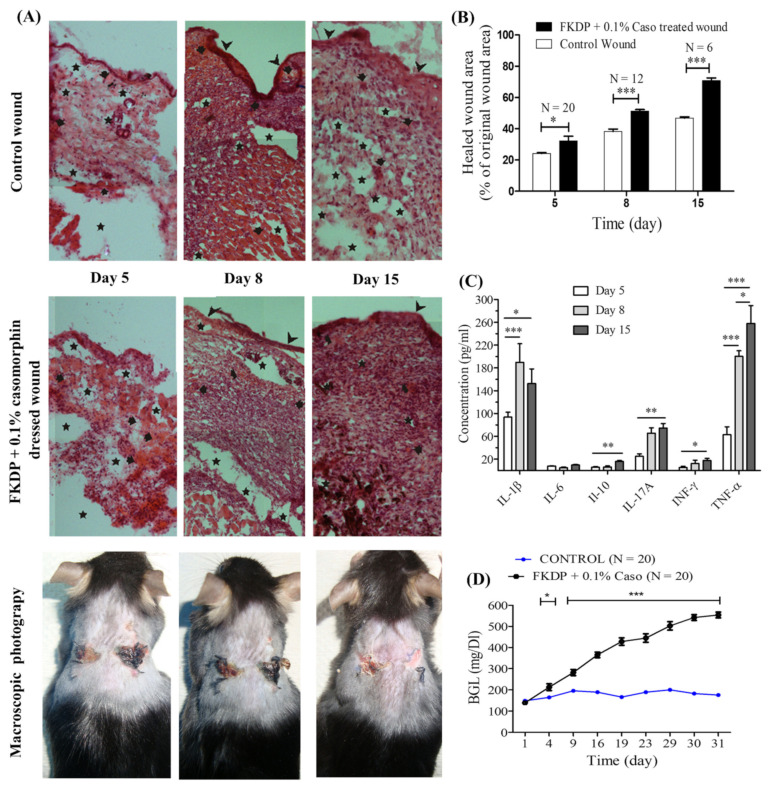
(**A**) Microscopic view of control and fur keratin-derived powder containing casomorphin (FKDP-0.1%Caso)-treated wounds during the course of healing; hematoxylin-eosin. Original magnification: 100×. Legend: 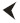-epidermis; 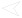-hair follicle; 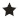-empty zones; 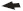-blood extravasation. (**B**) The effect of fur keratin-derived powder (FKDP) containing 0.1% casomorphin on skin wound healing. Because of the decreasing number of surviving mice, the data were only tested by the t-test for dependent samples (for each post-wounding day separately), with no prior two-way analysis of variance. Mice numbers at Day 5, N = 20; Day 8, N = 12; Day 15, N = 6. (**C**) Changes in cytokine level during wound healing in diabetic mice (*p*-value < 0.05 was considered significant, were * *p*-value < 0.05, ** *p*-value < 0.01, *** *p*-value < 0.001). (**D**) Changes in blood glucose level (BGL) during diabetes induction. (The data were statistically significant if *p* < 0.05 two-way analysis of variance, followed by Bonferroni post hoc tests, mean ± standard error of the mean) (color online, black and white in print).

It was observed that during the first 3 days after injection of streptozotocin (STZ) at the dose of 80 mg/kg or citrate buffer, the blood glucose level did not reach significant differences. From the fourth day of the experiment, an increase in blood glucose concentration was observed compared to the control group. The mice were considered diabetic when three consecutive measurements (29–31 days post-injection) showed elevated blood glucose levels > 250 mg/dL. In the control group (mice received only citrate buffer) the average blood glucose level was below 200 mg/dL during all experiment time points (Figure 2d). Before pharmacological induction of diabetes, body mass was measured. It was observed that mice lost weight after i.p. injection of STZ. In the previous studies, it was shown that healthy mice gained weight at the same time [20]. It should be mentioned animals had unlimited access to food and tap water, suggesting that weight loss was not affected by these factors. The health of the animals was monitored every day throughout the experiment. It was observed that during the development of diabetes changes in fur pigmentation was observed in mice given streptozotocin. At the beginning of the study, the fur of each animal was shiny, but due to the development of diabetes symptoms, the fur lost its shininess and became matt.

### 2.4. Influence of Examined Dressing on Skin Wound Healing in Diabetic Mice

The rate of wound healing was evaluated as the difference between initial wound area and area on each post-wounding day and expressed as a percent of the initial wound area according to the formula:

(1) $$\mathrm{Healed}\;\mathrm{wound}\;\mathrm{area}\;\mathrm{on}\;\mathrm{the}\;\mathrm{day}\;“i”\;=\frac{A_{0}-A_{i}}{A_{0}}\times 100\%$$

where *A*_0_ was the initial wound area and Ai was the unhealed wound area on the experiment day “i” (day 5, 8, or 15).

It was shown that wounds treated with a keratin dressing with 0.1% casomorphin healed significantly faster on the 5th, 8th, and 15th day of the experiment compared to untreated wounds (Figure 2b). On the fourth day after the injury, the percentage of healing for the treated wound was 32.30%, and for the untreated wound 24.10%. One week after the injury, statistically significant differences in the speed of wound healing were observed (the percentage of healing of the provided wound was 51.25%, while the untreated wound was 38.25%). Comparing the percentage of wound healing on day 14, it was found that the dressed wound was 70.75 % healed and the not-treated wound healed in 46.83%. It can be concluded that the addition of 0.1% casomorphin as a surface modifier significantly accelerated the healing process. This is most likely an example of the synergistic effect of both FKDP and casomorphin on the healing process.

### 2.5. Changes in Cytokine Level during the Healing Course

Cytokine level was measured during the healing course (Figure 2c). There were statistically significant differences in the concentration of IL-1β, IL-10, IL-17A, INF-γ, and TNF-α. In the case of IL-1β, the highest concentration was observed 8 days post-injury compared to day 5 (*p* 0.001) and day 15 (*p* < 0.05) respectively. An increased concentration of TNF-α was observed during recovery. Statistical significance was observed between day 5 vs. day 8 (*p* < 0.001), day 8 vs. day 15 (*p* < 0.05), and day 5 vs. day 15 (*p* < 0.001) respectively. Also statistically significant differences were observed in the concentrations of IL-10 and IL-17A (day 5 vs. day 15, *p* < 0.01) during the study period. Similar observations were found for INF-γ (day 5 vs. day 15, *p* < 0.05). There was no statistical significance in the concentration of IL-6 during wound healing.

### 2.6. Histopathological Studies

#### 2.6.1. Oozing and Microhemorrhages

During the first 24 h after injury, no increased exudate from the wound covered with keratin dressing containing casomorphin was observed. It was found that wounds covered with a keratin dressing showed no symptoms of redness, swelling, and oozing, which could indicate an inflammatory reaction to the applied dressing. After 24 h both wounds were covered with crust and no sign of exudate was observed.

In the next step, the impact of applied dressing on the number of microhemorrhages during wound healing was analyzed (Table 2). Five days post-injury a higher number of hemorrhages was noted in a dressed wound. Starting from day 5, a decreased number of microhemorrhages was observed on the dressed side in comparison to the undressed wound. Two weeks post-injury statistically significant number of microhemorrhages was observed in wounds treated with experimental dressing compare with control wound (*p* < 0.05).

#### 2.6.2. The Healing Course

During tissue recovery epidermis status was analyzed. Five days after surgery the epidermis was not restored in both wounds. The dressed wound was characterized by a slightly greater weaving of fibroblasts and a small number of voids than the untreated wound.

Seven days after the surgical procedure, the untreated wound was characterized by a thicker layer of the dermis than the treated wound (Figure 2a). The epidermis was restored in both wounds. Both wounds were characterized by the presence of voids and disturbances in the arrangement of the skin layers. During the healing process, the applied dressing was embedded in the wound at various depths. In its vicinity, places with a significant density of fibroblasts were observed.

Two weeks after surgical intervention, the treated wound showed a much higher cellular density in comparison to the untreated wound. In both wounds, a layer of the restored epidermis was visible. In the treated wound, the dermal layer showed the correct structure (similar to the healthy tissue). The tested dressing was embedded in the wound at various depths (Figure 2a). A significant concentration of fibroblasts was observed in the vicinity of the keratin dressing. In both wounds, there were a few places where the process of remodeling the regenerating tissue continued. In the untreated wound, there were single areas with extravasated erythrocytes into the wound lumen, and the assessed tissue was characterized by significant discontinuities and vortices. Both wounds completely regrow hair.

The applied FKDP + 0.1%Caso dressing reduced the tension of the tissue during the healing process, which influenced the scarring process. The resulting scar was practically invisible compared to the scar in the control wound. At this stage of research, it can be concluded that a combination of keratin dressing containing casomorphin as a surface modifier, significantly accelerated the healing process. This is most likely an example of the synergy between FKDP and casomorphin in the healing process.

#### 2.6.3. Cell Infiltrate into the FKDP-0.1%Caso and the Control Wounds

**Figure 3:**
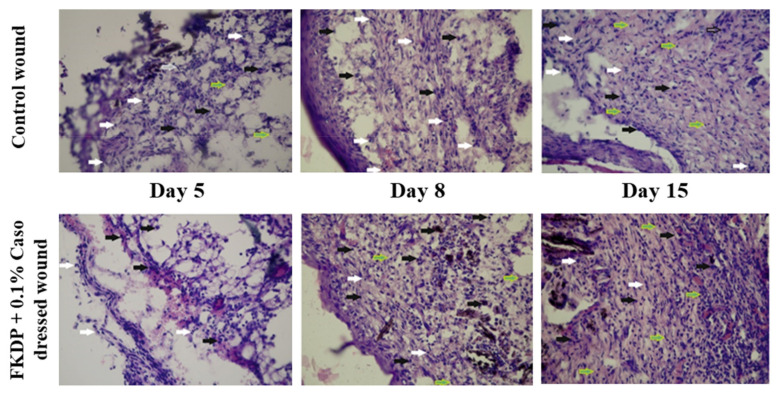
Microscopic view of the control and FKDP-0.1%casomorphin-treated wounds during the healing process. H&E. white arrow-neutrophils, black arrow-macrophages, green arrow-multinucleated giant cells, black and white arrow-lymphocytes; magnification 100× (color online, black and white in print).

During wound healing, the type of cells infiltrating the treated and untreated wounds was analyzed. It was observed that five days post-injury in FKDP + 0.1%Caso dressed wound the macrophage-neutrophilic reaction was predominant, wherein the undressed side prevailed neutrophilic (Table 3, Figure 3).

Nearby keratin dressing, scattered histiocytes, and lymphocytes were visible. The presence of these types of cells is favorable for the recovery process over the degenerative one present in the control wound.

On day eight, in the control wound neutrophils predominated with occasionally visible histiocytes. On the dressed side, histiocytes with the addition of foreign-body giant cells around the keratin dressing were observed. Two weeks post-injury in dressed side histiocytes, foreign-body giant cells, and lymphocytes were seen. The presence of these cells is responsible for better tissue remodeling and a proper healing process. In control wounds, a mixed infiltrate of neutrophils and macrophages was observed.

#### 2.6.4. Tissue Remodeling

**Figure 4:**
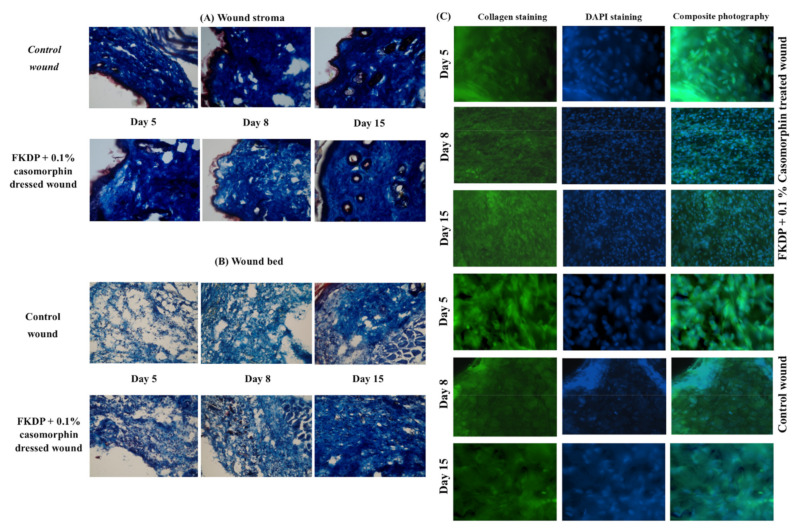
Masson Trichrome staining tissue derived from (**A**) wound stroma and (**B**) wound bed from control and FKDP + 0.1%Caso-treated wounds in 100× magnification. (**C**) Tissue biopsy taken from FKDP + 0.1%Caso (3 upper panels) treated wound and control wound (lower panel) immunolabeled for collagen (green), nucleus (blue), (color online, black & white in print).

Masson’s trichrome staining was used for visualization of collagen in skin biopsy specimens (Figure 4A,B). It was observed the stroma of the control wound was characterized by a denser weaving of collagen fibers. In the damaged tissue, single, thin, irregularly arranged collagen fibers were visible. In the dressed part, a loosening of the stromal structure with less frequently packed collagen fibers was observed. This finding indicates better conditions for the reconstruction of damaged and surrounding tissue in the initial phase of healing.

On the eighth day after surgery, it can be stated that the stroma of the wound with the tested dressing was characterized by the relaxation of collagen fibers. This means that the tissue is rebuilt in the wound, indicating better healing and stimulation of the synthesis of new collagen fibers. In the treated wound, a denser weaving of collagen fibers was observed in comparison to the untreated wound. Besides, the collagen fibers in the dressing wounds were thicker than in the untreated wound.

Two weeks later, the collagen fibers were markedly disorganized, they were thinner than in wounds treated with keratin dressing. In the dressed wound, the collagen fibers were thicker and better organized in the regenerated tissue.

### 2.7. Immunohistochemistry Staining

**Figure 5:**
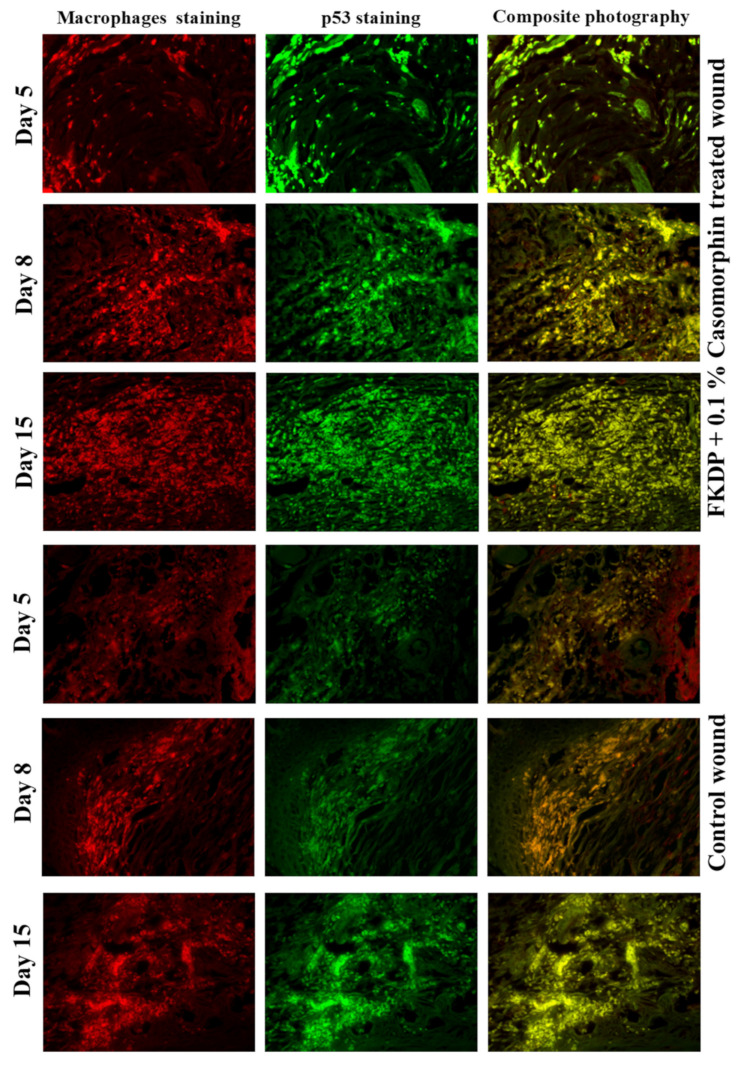
Tissue biopsy taken from FKDP + 0.1%Caso (3 upper panels) treated wound and control wound (lower panel) immunolabeled for macrophages (red), *p*53 (green). Note the changes in immunoreactive cell numbers during the healing course (color online, black & white in print).

In this study, immunohistochemical staining was performed for detection of macrophages, transcription factor NF-κB (*p*50 and *p*65 peptides), collagen fibers, and transcription factor p53 was performed. Cell nuclei were counterstained with bisbenzimide (in the routine Hoechst staining). In the control wounds, only a slight induction of macrophages was observed (Figure 5, lower panel). The number of these cells increased in the next post-injured time points (8, 15 days). All stained macrophages were positive also for *p*53. However, on the untreated side, one could find a region, with occasionally visible positive staining macrophages.

In the dressed wound, starting from the 5th-day post-surgery, the increased induction of immunostained macrophages was detected (Figure 5, upper panel). During the recovery time, it was observed that infiltration of macrophages was favorable for healing and correlated with hematoxylin and eosin staining. All cells were positive also for *p*53.

**Figure 6:**
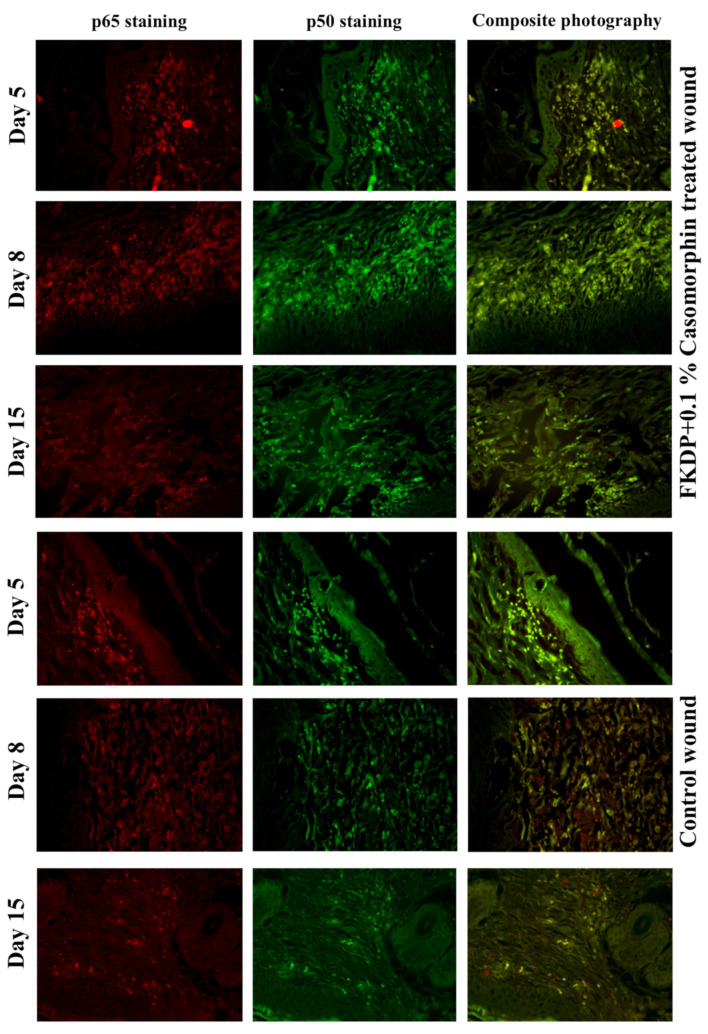
Tissue biopsy taken from FKDP + 0.1%Caso (3 upper panels) treated wound and control wound (lower panel) immunolabeled for *p*65 (red), *p*50 (green). Note the changes in immunoreactive cell numbers during the healing course (color online, black & white in print).

In the next step, we examined nuclear factor-kappa β (NF-κβ) which is associated with cell proliferation, adhesion, and inflammation. We observed that in dressed wounds during wound healing the immunolabelling for NF-κβ increased. Similar results were observed in control wounds; however, the immunolabeling was weaker when compared with a treated side (Figure 6).

During the recovery process, we also examined collagen remodeling in the control and dressed wounds. The immunohistochemical examination confirmed results observed in Masson-Trichrome staining (Figure 4A–C). We observed that collagen fibers were more regularly positioned on the dressed side when compared to the undressed wound. Also, higher nuclear density was observed in FKDP-0.1%Caso dressed the wound. It could be speculated that keratin dressing stimulated cell activation and migration when compared to untreated sides, where the reconstruction process had weaker intensity.

## 3. Discussion

In the current study, we investigated a novel therapeutic approach to wound healing-a wound dressing based on fur keratin-derived powder enriched with casomorphin. A new finding of our study is that obtained dressing containing the opioid peptide-casomorphin supported skin wound healing and increased macrophages infiltration into the wound, which could shorten the inflammatory phase, induce faster tissue remodeling, and thus accelerate healing.

Diabetes mellitus is a major medical problem that can cause impaired wound healing by affecting one or more biological mechanisms involved in this process. It is a major medical problem, probably triggered by hyperglycemia, chronic inflammation, micro-, and macroangiopathy, and nerve damage, which can induce the sensation of pain [1]. At the physiological level, wound pain originates from tissue damage or nervous system dysfunction. Chronic wound pain may originate from both nociceptive and neuropathic elements [21]. Bechert and Abraham [22] reported that pain is often an overlooked factor in wound care and wound healing that affects wound care practice, and the nature of pain which is experienced by the patient is directly related to the type of wound sustained. Opioids are routinely used as analgesics in patients with chronic wounds; however, the impact of opioid exposure on wound healing is poorly understood [23]. The development of efficient wound dressings for chronic, non-healing wounds is a substantial challenge.

Keratin biomaterials soluble or insoluble are promising candidates in the field of wound dressing materials due to their tissue biocompatibility and ability to support cell growth [8,20]. Moreover, several studies have shown that insoluble keratin enhances tissue recovery and promotes macrophages infiltration during the skin repair process in healthy and diabetic animals. The potentially extensive surface allows modification of compounds with antibacterial or analgesic properties, which is an additional advantage [20,24,25].

Capillary electrophoresis (CE) has great potential for the analysis of compounds of pharmaceutical interest such as anti-inflammatory drugs, opiates, benzodiazepines, and alkaloids [26,27]. CE allows fast, low-cost, high separation efficiency, and low consumption of reagents and samples [8,28]. In this study, CE was applied for the detection of released casomorphin from the experimental keratin dressing. It was observed that casomorphin was slowly released from keratin dressing during 5 consecutive days. It should be mentioned that this is one of the first applications of capillary electrophoresis to assess the release of such biologically active drugs from the dressing.

In the next step, we evaluated the effect of morphine and casomorphin on cell viability and cell migration using an in vitro scratch assay protocol. However, there are limited studies about the effect of casomorphin on cell proliferation and wound healing. We found various information about the effects of morphine on cell growth and proliferation. In this study, we did not observe any toxic effect of morphine and casomorphin on cell viability. Nishiwada et al. [29] demonstrated that morphine exposure reduced cell viability and enhanced cytotoxicity in HSC-3 cells in a concentration-dependent manner. Similar results were observed by Kamp et al. [30] in the case of casomorphin peptides which decrease the proliferation of prostatic cancer cell lines (LNCaP, PC3, and DU145). In our study, it was shown that casomorphin in concentration 100 µM increases cell viability. In the next step, we evaluated the influence of keratin dressing and keratin opioid-loaded dressing on cell viability. We showed that after 24 h incubation, keratin-containing examined opioids increased cell growth compared with controls. It was shown that cells incubated with keratin containing casomorphin had higher viability after 48 h incubation. The obtained results documented that keratin dressing is nontoxic and enhances cell proliferation, as was shown by Bochyńska et al. [7].

During the healing process cytokine levels were measured on days 5, 8, and 15 post-injury. The level of IL-6, IL-10, IL-17A, IFN-γ, and TNF-α was detected on each day after the injury. We observed a statistically significant decrease in the levels of pro-inflammatory cytokines such as IL-1β, IL-17A, IFN-γ, and TNF-α; however, we did not detect statistically significant differences in the level of IL-6. On the contrary, the level of anti-inflammatory cytokine IL-10 increased during wound healing.

IL-1β is a polypeptide that is produced by macrophages, fibroblasts, and neutrophils in response to invasive effects such as infection or injury. It has been documented in several reports that IL-1β is up-regulated during wound healing. However, there is no consensus or direct evidence that IL-1β activity plays a central role in the healing process [31].

The concentration of IL-1β increased during the recovery process compared to day 5. The highest concentration of IL-1β was observed on day 8 post-injury. It has been reported that levels of the pro-inflammatory cytokines IL-1 and TNF-α are higher in non-healing wounds compared with healing wounds, as when fibroblasts become nonresponsive, cytokine secretion due to neutrophil and macrophage activity increases as a feedback mechanism to stimulate a response from the fibroblast cells. These cytokine levels dropped significantly when the healing began to occur [32,33].

During the healing course, we observed an increased level of IL-10, which can be associated with increased infiltration of macrophages, multinucleate giant cells (MNGCs) and histiocytes, and a decreased infiltration with inflammatory cells (neutrophils and lymphocytes). IL-10 is involved in macrophage activation and switches the M1 phenotype into M2 responsible for inflammation quenching and tissue remodeling [34]. The enhanced level of IL-10 correlates with enlarged infiltration with macrophages during healing which was confirmed using histology and immunohistochemistry.

Interleukin-17 activates many signaling cascades and production of another cytokine such as IL-6, TNF-α, IL-1β and is also involved in the induction of pro-inflammatory responses [35,36,37]. In our experiment, the level of IL-17 and IFN-γ significantly increased. IL-17A is required for efficient skin wound healing, as IL-17a−/− mice exhibit defects in wound repair [38]. An increased concentration of IFN-γ correlated with tissue response, where increased infiltration of macrophages was observed. It is a well-known role of IFN-γ-mediated polarization of macrophages to an ‘M1-like’ state, which is characterized by increased pro-inflammatory activity and macrophage resistance to tolerogenic and anti-inflammatory factors [39].

The transcription factor p53, activated by various cellular stress such as DNA damage or hypoxia, plays an important role in cutaneous wound healing [40]. It was observed that in FKDP + 0.1%Caso treated wounds, p53 immunolabeling was higher than in controls. The increased p53 expression in fibroblastic cells also corresponded to the time-dependent re-epithelialization of skin wounds [41]. During the wound healing, we also examined nuclear factor-κB (NF-κβ), which regulates a large array of genes involved in different processes of the immune and inflammatory responses and actively participates in wound healing [42]. The pro-inflammatory function of NF-κβ has been extensively studied in macrophages. Under different pathophysiologic conditions, activated macrophages can transform into the classically activated (M1) and the alternatively activated (M2) macrophages [43]. These cells perform various functions. M1 polarized macrophages are characterized by the production of pro-inflammatory cytokines such as IL-1, IL-6, TNF-α, which are involved in various inflammatory responses and promote the differentiation of inflammatory T lymphocytes. M2 macrophages, on the other hand, produce anti-inflammatory cytokines such as IL-10 and IL-13 and are important in resolving inflammation and mediating wound healing [43,44,45]. It is well known that NF-κβ is associated with cell proliferation, adhesion, inflammation, and elimination of reactive oxygen species [45]. In keratin-treated wounds, we observed increased fibroblast-rich cellular infiltrate near the keratin dressing when compared to control wounds, and we hypothesized that NF-κβ might regulate this aspect of the wound healing process. Several studies showed that NF-κβ signals play an important role in the healing of different wound types (corneal epithelial wound healing, scratch injury, and cutaneous wound healing) [45,46,47,48,49].

For the in vivo evaluation of the insoluble keratin dressing containing casomorphin (FKDP + 0.1%Caso) the full-thickness wound model in diabetic mice was employed. In wounds treated with keratin dressing, no signs of oozing were observed, which proves the sorption properties of the tested biomaterial. In the control wounds, a slight exudate was observed in 10% of the animals, which disappeared 24 h later. Based on our previous research and literature data, it is known that keratin-based biomaterials have sorption properties [8,50,51]. Wounds treated with a keratin dressing containing casomorphin healed significantly faster throughout the experiment when compared to the control wounds. Besides, the wound covered with keratin dressing containing casomorphin underwent epithelization faster than the untreated wounds. It was observed that the examined dressing not only facilitates epithelial cell migration and proliferation but also favors a moist environment advantageous for wound healing. The obtained results are in line with the previous study conducted on healthy and diabetic animals with surgical wounds, where wounds were dressed with an insoluble fraction of keratin biomaterials [20,52]. Similar results were observed by other researchers who examined the soluble fraction of keratin. They documented that wounds dressed with different keratin biomaterials underwent faster epithelization and did not show an inflammatory reaction [53,54,55].

Opioids, especially morphine, are routinely used as painkillers in patients with chronic wounds. However, the impact of opioid exposure on wound healing is poorly understood. Several studies have suggested that opioid use (morphine in particular) may disturb healing, by reducing immune activation, impairing tissue oxygenation and angiogenesis [56,57], and altering myofibroblast recruitment as well as impacting negatively upon the keratinocytes, cytokine production, endothelial proliferation, and angiogenesis [9,56,57].

Shanmugam et al. [1] suggested that opioid treatment is associated with poor healing in patients with chronic, non-healing wounds. In our studies, we showed that dressing containing casomorphin enhanced healing in diabetic conditions. Moreover, wounds covered with keratin dressing containing casomorphin underwent faster epithelization in comparison to the control wounds by facilitating epithelial cell migration and proliferation. On the dressed side macrophages, histiocytes, and lymphocytes predominated, rather than neutrophils as delineated in the control wound neutrophils. The presence of macrophages and their morphological variants favor tissue remodeling and better wound closure [8,58].

Poonawala et al. [19] examined the effect of topically applied opioids in cream (fentanyl, hydromorphone, and morphine) on the healing of open ischemic wounds in rats. Topically applied opioids hastened wound closure, particularly in the first 4 days when no healing was initiated in phosphate-buffered saline solution-treated wounds.

Detection of mechanisms supporting the wound healing process following the use of the insoluble keratin wound dressing containing casomorphin needs further intensive investigations. There is no data available on casomorphin role in wound healing either in vitro and in vivo, neither any information about cell signaling or molecular pathways activated by this compound. However, some articles indicate that casomorphin may protect the cell from death and increase cell viability. Zhang and colleagues [59] showed that casomorphin plays a protective role in the acute kidney injury (AKI) induced by sepsis. In their model, casomorphin inhibited the NF-κB activation and reduced oxidative stress and inflammation. In our model, we detected the neutrophil infiltrate reduction of the dressed wound and simultaneously the higher number of macrophages in the peri-wound area. Additionally, we observed an increase of NF-κB staining both in dressed as well as undressed wounds, but immunosignal up-regulation was lower in the latter. Verren et al. [60] showed the connection between the reduction of macrophage infiltrates and down-regulation of NF-κB. We postulate that NF-κB increases in the infiltration of the dressed area by macrophages. It is known that NF-κB plays an important role in many processes during tissue injury and healing. We suspect that its increase is connected with the induction of pro-survival action of this factor. Zhu and colleagues [61] proved the beneficial role of casomorphin in the oxidative stress of human lens epithelial cells (HLECs). Casomorphin increased the cell survival via induction of expression of Forkhead box o1 (Foxo1), SP1, and up-regulation of antioxidant enzymes such as superoxide dismutase (SOD) and glutathione peroxidase (GSH-px). It is known that injury may result in oxidative stress, so that kind of cell-protective mechanism or similar protection is also possible in our experimental model. Sakaguchi et al. [62] indicated that the protection of nervous cells by casomorphin may act via opioid receptors and G protein signaling pathway. These receptors are present on the C fibers localized in the dermis that transmit pain on the inflammatory cells.

In this examination, Masson’s trichrome staining for collagen visualization was used. We showed that in wounds covered with keratin powder and casomorphin, there was a significant increase in the formation of collagen fibers compared to control wounds. Several studies showed that an insoluble fraction of keratin stimulates the formation of novel collagen fibers [8,52]. Also, Poonawala et al. [19] observed an increased content of collagen fibers in wounds treated with opioid cream.

## 4. Materials and Methods

All technical details are described in detail in the Supplementary Materials.

### 4.1. Preparation of Experimental Wound Dressings

Fur keratin-derived powder (FKDP) was coated with 0.1% solution of casomorphin (Caso) was prepared as described previously [8]. The final product was labeled as FKDP + 0.1%Caso and was used as a wound dressing.

### 4.2. In Vitro Drug Release from the Wound Dressing

Samples of keratin dressing containing casomorphin (10 mg) were placed in a tube filled with 5 mL of sterile PBS (pH = 7.4). Then samples were placed in a shaker operating at 80 rpm and 37 °C. Liquid media (500 µL) were collected from each tube at specific time intervals (0.5 h, 1 h, 2 h, 3 h, 4 h, 24 h, 48 h, 72 h, 96 h, 120 h) and replaced with 500 µL of fresh medium to keep the volume constant. The absorbance of the samples was measured and determined with UV-VIS detection at 214 nm using a capillary electrophoresis system (CE).

### 4.3. Electrophoretic Measurements

Capillary electrophoresis analysis was performed using Agilent 7100 series CE instrument (Agilent Technologies, Santa Clara, CA, USA) equipped with a UV-VIS diode array detector and ChemStation software (AgilentOpenLABCDSChemStation version 1.7, Agilent Technologies, Santa Clara, CA, USA) for instrument control and data collection. The samples were e directly injected into the capillary electrophoresis system.

### 4.4. Cell Proliferation Assay

Cell Proliferation Kit I (MTT-3-(4,5-dimethylthiazol-2-yl)-2,5-diphenyltetrazolium bromide) (Cat. No. 11 465 007 001, Roche, Warsaw, Poland) was used to determine the viability of murine fibroblasts NIH/3T3 treated with experimental keratin dressings. The experiments were performed according to the manufacturer protocol.

### 4.5. In Vitro Wound-Healing Assay

A “scratch” was created in a monolayer of NIH/3T3 cells (ATCC, MA, USA). Cells were then incubated with experimental keratin dressings for up to 48 h. The rate of migration was calculated by measuring the distance between the edges of the wound at a specific time point.

### 4.6. Animals

Twenty 12–15-week-old male C57BL6/J mice (23–25 g of body weight) were acquired from the Mossakowski Medical Research Centre. Mice were housed in plastic cages in a group of five and allowed free access to food and water. They were maintained in a temperature-, humidity- and light-controlled environment with a 12 h light-dark cycle. After surgery (full-thickness skin wound model) the animals were housed individually. The experimental design of the study was approved by the 4th Local Ethics Committee for Experiments on Animals at the National Medicines Institute, Warsaw, Poland (Certificate of approval no. 58/2012). Procedures adhered to guidelines published in European Directive 2010/63/EU on the protection of animals used for scientific purposes.

### 4.7. Iatrogenically Induced Diabetes

Diabetes in mice (N = 20) was induced with 5 daily intraperitoneal (i.p.) injections of streptozotocin (80 mg/kg body weight) as described previously [52]. Mice from the control group (N = 20) received citrate buffer only. A mouse was considered diabetic if three consecutive measurements showed a blood glucose level higher than 250 mg/dL.

### 4.8. Surgical Procedure

The surgical procedure was performed in general anesthesia according to the method described by Konop et al. [8,20,52]. The fur was shaved, the skin was disinfected with 70% ethanol and two full-thickness skin wound of 10 mm diameter each were made on the mice’s back. The FKDP + 0.1%Caso dressing was applied once to one wound on the right side with care to fully and evenly cover the wound without masking its edges. The second wound served as a control and remained undressed. Wounds were photographed at each time point (day, 5, 8, and 15 post-injury), 6–8 mice were sacrificed by cervical dislocation and skin biopsy with a 1 to 2 mm margin were taken for histopathological examination.

### 4.9. Histopathological Analysis

Skin samples were prepared accordingly to pathological standards described in detail in the Supplementary Materials. Routine hematoxylin and eosin staining was performed. To determine the collagen deposition, a Masson-Trichrome staining kit (Diapath S.p.A. Martinengo Italy) was used. To determine blood extravasation (microhemorrhages), six H&E-stained sections from each mouse from the control and FKDP + 0.1%Caso wounds for each time point were used. Only sections containing the full extent of the wound were chosen. The mean number of microhemorrhages for each wound was calculated for statistical analysis. The sections were examined using an Eclipse Ni-U Nikon (Nicon Instruments Inc., Toki, Japan) light microscope.

### 4.10. Cytokine Analysis

Blood samples were taken on Days 5, 8, and 15 post-injury. Measurement of serum concentration of interleukin (IL)-1β, IL-6, IL-10, IL-17A, interferon-gamma (IFN-γ), and tumor necrosis factor-alpha (TNF-α) was performed using Bio-Plex Pro Mouse Cytokine Panel (lot #5030291, Bio-Rad Laboratories, Hercules, CA, USA) according to the manufacturer’s protocol. Magnetic beads were analyzed at a Bio-Plex 200 (Luminex, Bio-Rad Bio-Rad Laboratories, Hercules, CA, USA). Cytokines concentration is expressed in pg/mL.

### 4.11. Immunohistochemistry Staining

Immunohistochemical staining for macrophages, Nuclear Factor kappa B (subunits: p65 and p50), and collagen IV were performed with the appropriate primary and secondary antibodies. Immunostaining was detected with a model Optiphot-2 Nikon fluorescent microscope (Nicon Instruments Inc., Toki, Japan) equipped with the appropriate filters and recorded with a model DS-L1 Nikon camera (Nicon Instruments Inc., Toki, Japan).

### 4.12. Statistical Analysis

All the data are presented as means ± SEM. We performed all the analyses using two-way ANOVA followed by the Bonferroni posthoc test to calculate the differences between the groups. Student’s *t*-tests for analysis of dependent and independent variables were also used. In all cases, a *p*-value of <0.05 was considered significant. All comparisons were calculated using GraphPad Prism 5.0 software for Windows (GraphPad Software, San Diego, CA, USA).

## 5. Conclusions

In summary, our study showed that keratin dressing supplemented with casomorphin is safe and efficient, promoting skin wound healing in diabetic mice. We have proven that casomorphin was slowly released from the dressing, and in vitro and in vivo studies documented that keratin dressing is biocompatible, supports cell growth, and provides an immunomodulatory effect. Moreover, it stimulated macrophages infiltration which favors remodeling and tissue regeneration. The dressing was naturally incorporated in regenerated tissue during the wound healing process. Applied keratin dressing favored reconstruction of more regular skin structure and assured enhanced cosmesis effect in terms of scar formation and final appearance. However, future studies are needed to better understand the molecular mechanism involved in skin repair induced by keratin scaffolding, especially the role of keratin 16 and 17 in skin wound healing.

## Figures and Tables

**Table 1:** Semi-quantitative results of the CE study: detected concentration of casomorphin released from keratin dressing into PBS measured during 120 h.

The Concentration of Released Casomorphin FKDP + 0.1%Caso Dressing into PBS [mmol/mL] (C ± SD)									
0.5 h	1 h	2 h	3 h	4 h	24 h	48 h	72 h	96 h	120 h
0.628	0.523	0.508	0.520	0.526	0.599	0.591	0.567	0.569	0.599
± 0.103	± 0.023	± 0.033	± 0.024	± 0.043	± 0.053	± 0.058	± 0.058	± 0.081	± 0.122

**Table 2:** The effect of FKDP-casomorphin dressing on the epidermis and the time course of changes in the number of microhemorrhages in full-thickness surgical skin wounds in male C57BL/6J mice.

Experiment Day (Number of Mice)	Wound Status	Epidermal Status	Number of Micro-BloodExtravasations (mean ± SD)	ANOVA Results
Day 5 (N = 3)	Undressed	−	16.56 ± 3.87	Experimental day effect: F_2.6_ = 139.10 *p* = 0.001 Dressing effect: F_1.6_ = 0.65 *p* = 0.452 Interaction effect (Dressing × Experiment Day) F_2.6_ = 1.81 *p* = 0.242
	Dressed	−	19.00 ± 2.19	
Day 8 (N = 3)	Undressed	+	14.39 ± 2.10	
	Dressed	+	11.17 ± 1.86	
Day 15 (N = 3)	Undressed	+	6.39 ± 0.84	
	Dressed	++	4.00 ± 0.33 **^,#^	

**-*p* < 0.01 relative to the corresponding value for a dressed wound on day 5, 8, #-*p* < 0.05 relative to the corresponding value for the control wound; Abbreviations: −: ANOVA: analysis of variance; no visible epidermis; +: thin epidermis covering most of the wound; ++: epidermis of increased thickness covering the entire wound.

**Table 3:** Differences in cell types detected in tissues derived from the control and dressed full-thickness surgical skin wounds in male C57BL/6J mice.

		Cells Tyee			
		Neutrophils	Macrophages		Lymphocytes
			Histiocytes	Foreign-Body Giant Cell (FBGC)	
5D (N = 3)	Control wound	+++	+	−	+
	Dressed wound	++	++	+	−
8D (N = 3)	Control wound	+++	+	−	+
	Dressed wound	+	++	+	+
15D (N = 3)	Control wound	++	++	+	++
	Dressed wound	+	++	+++	++

Note. Cells detected in the wound bed (100 cells per field of view) under magnification 400×: −, no visible cells; +, occasionally visible cells (0–20%); +, low cell number (20–50%); +++, medium cell abundance (50–70%); ++++, high cell abundance (70–100%).

## Data Availability

Not applicable.

## Supplementary Materials

Figure S1: CE electropherogram obtained for casomorphin released from keratin dressing, Figure S2: Effect of opioids on cell viability after 24 and 48 h, Figure S3: Effect of opioids on cell migration after 24 and 48 h, Figure S4: The rate of wound closure in NIH/3T3 monolayer treated with opioids, Figure S5: The rate of wound closure in NIH/3T3 monolayer treated with experimental dressings.

## References

[1] Shanmugam V.K., Couch K.S., McNish S., Amdur R.L. (2017). Relationship between opioid treatment and rate of healing in chronic wounds. Wound Repair Regen..

[2] Lesniak A., Bochynska-Czyz M., Sacharczuk M., Benhye S., Misicka A., Bujalska-Zadrożny M., Lipkowski A.W. (2016). Biphalin preferentially recruits peripheral opioid receptors to facilitate analgesia in a mouse model of cancer pain—A comparison with morphine. Eur. J. Pharm. Sci..

[3] Shavandi A., Silva T.H., Bekhit A.A., Bekhit A.E.-D.A. (2017). Keratin: Dissolution, extraction and biomedical application. Biomater. Sci..

[4] Lin C.-W., Chen Y.-K., Tang K.-C., Yang K.-C., Cheng N.-C., Yu J. (2019). Keratin scaffolds with human adipose stem cells: Physical and biological effects toward wound healing. J. Tissue Eng. Regen. Med..

[5] Feroz S., Muhammad N., Ratnayake J., Dias G. (2020). Keratin-based materials for biomedical applications. Bioact. Mater..

[6] Rajabi M., Ali A., McConnell M., Cabral J. (2020). Keratinous materials: Structures and functions in biomedical applications. Mater. Sci. Eng. C.

[7] Bochynska-Czyz M., Redkiewicz P., Kozlowska H., Matalinska J., Konop M., Kosson P. (2020). Can keratin scaffolds be used for creating three-dimensional cell cultures?. Open Med..

[8] Konop M., Czuwara J., Kłodzińska E., Laskowska A.K., Sulejczak D., Damps T., Zielenkiewicz U., Brzozowska I., Sureda A., Kowalkowski T. (2020). Evaluation of keratin biomaterial containing silver nanoparticles as a potential wound dressing in full-thickness skin wound model in diabetic mice. J. Tissue Eng. Regen. Med..

[9] Rook J.M., Hasan W., McCarson K.E. (2009). Morphine-induced early delays in wound closure: Involvement of sensory neuropeptides and modification of neurokinin receptor expression. Biochem. Pharmacol..

[10] Flock P. (2003). Pilot study to determine the effectiveness of diamorphine gel to control pressure ulcer pain. J. Pain Symptom Manag..

[11] Long T.D., Cathers T.A., Twillman R., O’Donnell T., Garrigues N., Jones T. (2001). Morphine-Infused Silver Sulfadiazine (MISS) cream for burn analgesia: A pilot study. J. Burn. Care Rehabil..

[12] Cerchietti L.C.A., Navigante A.H., Bonomi M.R., Zaderajko M.A., Menéndez P.R., Pogany C.E., Roth B.M.C. (2002). Effect of topical morphine for mucositis-associated pain following concomitant chemoradiotherapy for head and neck carcinoma. Cancer.

[13] Thiruvengadam M., Venkidasamy B., Thirupathi P., Chung I.-M., Subramanian U. (2021). β-Casomorphin: A complete health perspective. Food Chem..

[14] Zhang W., Song S., Liu F., Liu Y., Zhang Y. (2015). Beta-casomorphin-7 prevents epithelial-mesenchymal transdifferentiation of NRK-52E cells at high glucose level: Involvement of AngII-TGF-β1 pathway. Peptides.

[15] Chang W.H., Zheng A.J., Chen Z.M., Zhang S., Cai H.Y., Liu G.H. (2019). β-Casomorphin increases fat deposition in broiler chickens by modulating expression of lipid metabolism genes. Animal.

[16] Zhang W., Miao J., Wang S., Zhang Y. (2013). The protective effects of beta-casomorphin-7 against glucose-induced renal oxidative stress in vivo and vitro. PLoS ONE.

[17] Yin H., Miao J., Zhang Y. (2010). Protective effect of β-casomorphin-7 on type 1 diabetes rats induced with streptozotocin. Peptides.

[18] Rook J.M., McCarson K.E. (2007). Delay of cutaneous wound closure by morphine via local blockade of peripheral tachykinin release. Biochem. Pharmacol..

[19] Poonawala T., Levay-Young B.K., Hebbel R.P., Gupta K. (2005). Opioids heal ischemic wounds in the rat. Wound Repair Regen..

[20] Konop M., Czuwara J., Kłodzińska E., Laskowska A.K., Zielenkiewicz U., Brzozowska I., Nabavi S.M., Rudnicka L. (2018). Development of a novel keratin dressing which accelerates full-thickness skin wound healing in diabetic mice: In vitro and in vivo studies. J. Biomater. Appl..

[21] Stein C., Küchler S. (2013). Targeting inflammation and wound healing by opioids. Trends Pharmacol. Sci..

[22] Bechert K., Abraham S.E. (2009). Pain management and wound care. J. Am. Coll. Certif. Wound Spec..

[23] Stein C. (2012). Non-analgesic effects of opioids: Peripheral opioid effects on inflammation and wound healing. Curr. Pharm. Des..

[24] Konop M., Damps T., Misicka A., Rudnicka L. (2016). Certain aspects of silver and silver nanoparticles in wound care: A minireview. J. Nanomater..

[25] Muchowska A., Redkiewicz P., Różycki K., Matalińska J., Lipiński P.F., Czuwara J., Kosson P. (2019). The analgesic hybrid of dermorphin/substance P and analog of enkephalin improve wound healing in streptozotocin-induced diabetic rats. Wound Repair Regen..

[26] Taylor R., Low A., Reid R. (1996). Determination of opiates in urine by capillary electrophoresis. J. Chromatogr. B Biomed. Sci. Appl..

[27] Ding Y., Garcia C.D. (2006). Determination of nonsteroidal anti-inflammatory drugs in serum by microchip capillary electrophoresis with electrochemical detection. Electroanalysis.

[28] Cui X., Ni C., Liang C., Gong F., Wang R., Chen G., Zhang Y. (2019). Screening and quantitation of forty-six drugs of abuse and toxic compounds in human whole blood by capillary electrophoresis: Application to forensic cases. Microchem. J..

[29] Nishiwada T., Kawaraguchi Y., Uemura K., Kawaguchi M. (2019). Morphine inhibits cell viability and growth via suppression of vascular endothelial growth factor in human oral cancer HSC-3 cells. J. Anesthesia.

[30] Kampa M., Bakogeorgou E., Hatzoglou A., Damianaki A., Martin P.-M., Castanas E. (1997). Opioid alkaloids and casomorphin peptides decrease the proliferation of prostatic cancer cell lines (LNCaP, PC3 and DU145) through a partial interaction with opioid receptors. Eur. J. Pharmacol..

[31] Chan A.H., Schroder K. (2020). Inflammasome signaling and regulation of interleukin-1 family cytokines. J. Exp. Med..

[32] Yussof S.J.M., Omar E., Pai D.R., Sood S. (2012). Cellular events and biomarkers of wound healing. Indian J. Plast. Surg..

[33] Trengove N.J., Bielefeldt-Ohmann H., Stacey M.C. (2001). Mitogenic activity and cytokine levels in non-healing and healing chronic leg ulcers. Wound Repair Regen..

[34] Shapouri-Moghaddam A., Mohammadian S., Vazini H., Taghadosi M., Esmaeili S.-A., Mardani F., Seifi B., Mohammadi A., Afshari J.T., Sahebkar A. (2018). Macrophage plasticity, polarization, and function in health and disease. J. Cell. Physiol..

[35] Monin L., Gaffen S.L. (2018). Interleukin 17 family cytokines: Signaling mechanisms, biological activities, and therapeutic implications. Cold Spring Harb. Perspect. Biol..

[36] Li Y., Wu J., Luo G., He W. (2018). Functions of Vγ4 T cells and dendritic epidermal t cells on skin wound healing. Front. Immunol..

[37] Akitsu A., Iwakura Y. (2018). Interleukin-17-producing γδ T (γδ17) cells in inflammatory diseases. Immunology.

[38] MacLeod A.S., Hemmers S., Garijo O., Chabod M., Mowen K., Witherden D.A., Havran W.L. (2013). Dendritic epidermal T cells regulate skin antimicrobial barrier function. J. Clin. Investig..

[39] Greenlee-Wacker M.C., Nauseef W.M. (2016). IFN-γ targets macrophage-mediated immune responses toward Staphylococcus aureus. J. Leukoc. Biol..

[40] Pan S.-C., Li C.-Y., Kuo C.-Y., Kuo Y.-Z., Fang W.-Y., Huang Y.-H., Hsieh T.-C., Kao H.-Y., Kuo Y., Kang Y.-R. (2018). The p53-S100A2 positive feedback loop negatively regulates epithelialization in cutaneous wound healing. Sci. Rep..

[41] Hausmann R., Nerlich A., Betz P. (1998). The time-related expression of p53 protein in human skin wounds—A quantitative immunohistochemical analysis. Int. J. Leg. Med..

[42] Liu T., Zhang L., Joo D., Sun S.-C. (2017). NF-κB signaling in inflammation. Signal Transduct. Target. Ther..

[43] Wang N., Liang H., Zen K. (2014). Molecular mechanisms that influence the macrophage M1–M2 polarization balance. Front. Immunol..

[44] Mosser D.M. (2003). The many faces of macrophage activation. J. Leukoc. Biol..

[45] Park Y.R., Sultan T., Park H.J., Lee J.M., Ju H.W., Lee O.J., Lee D.J., Kaplan D.L., Park C.H. (2018). NF-κB signaling is key in the wound healing processes of silk fibroin. Acta Biomater..

[46] Gurtner G.C., Werner S., Barrandon Y., Longaker M.T. (2008). Wound repair and regeneration. Nat. Cell Biol..

[47] Chen J., Chen Y., Chen Y., Yang Z., You B., Ruan Y.C., Peng Y. (2016). Epidermal CFTR suppresses MAPK/NF-κB to promote cutaneous wound healing. Cell. Physiol. Biochem..

[48] Wang L., Wu X., Shi T., Lu L. (2013). Epidermal Growth Factor (EGF)-induced corneal epithelial wound healing through nuclear factor κB subtype-regulated CCCTC binding factor (CTCF) activation. J. Biol. Chem..

[49] Heo S.C., Jeon E.S., Lee I.H., Kim H.S., Kim M.B., Kim J.H. (2011). Tumor necrosis factor-α-activated human adipose tissue-derived mesenchymal stem cells accelerate cutaneous wound healing through paracrine mechanisms. J. Investig. Dermatol..

[50] Stęplewski W., Wawro D., Ratajska M., Wrześniewska-Tosik K. (2007). Novel biocomposites with feather keratin. Fibres Text. East. Eur..

[51] Katoh K., Shibayama M., Tanabe T., Yamauchi K. (2003). Preparation and properties of keratin-poly(vinyl alcohol) blend fiber. J. Appl. Polym. Sci..

[52] Konop M., Sulejczak D., Czuwara J., Kosson P., Misicka A., Lipkowski A.W., Rudnicka L. (2017). The role of allogenic keratin-derived dressing in wound healing in a mouse model. Wound Repair Regen..

[53] Yuan J., Geng J., Xing Z., Shim K.-J., Han I., Kim J.-C., Kang I.-K., Shen J. (2015). Novel wound dressing based on nanofibrous PHBV-keratin mats. J. Tissue Eng. Regen. Med..

[54] Shanmugasundaram O., Ahmed K.S.Z., Sujatha K., Ponnmurugan P., Srivastava A., Ramesh R., Sukumar R., Elanithi K. (2018). Fabrication and characterization of chicken feather keratin/polysaccharides blended polymer coated nonwoven dressing materials for wound healing applications. Mater. Sci. Eng. C.

[55] Veerasubramanian P.K., Thangavel P., Kannan R., Chakraborty S., Ramachandran B., Suguna V.L. (2018). Muthuvijayan, Corrigendum to “An investigation of konjac glucomannan-keratin hydrogel scaffold loaded with Avena sativa extracts for diabetic wound healing”. Colloids Surf. B Biointerfaces.

[56] Martin J.L., Charboneau R., Barke R.A., Roy S. (2010). Chronic morphine treatment inhibits LPS-induced angiogenesis: Implications in wound healing. Cell. Immunol..

[57] Martin J.L., Koodie L., Krishnan A.G., Charboneau R., Barke R.A., Roy S. (2010). Chronic morphine administration delays wound healing by inhibiting immune cell recruitment to the wound site. Am. J. Pathol..

[58] Habiboallah G., Mahdi Z., Majid Z., Nasroallah S., Taghavi A.M., Forouzanfar A., Arjmand N. (2014). Enhancement of gingival wound healing by local application of silver nanoparticles periodontal dressing following surgery: A histological assessment in animal model. Mod. Res. Inflamm..

[59] Zhang Z., Zhao H., Ge D., Wang S., Qi B. (2019). β-casomorphin-7 ameliorates sepsis-induced acute kidney injury by targeting NF-κB pathway. Med Sci. Monit..

[60] Veeren B., Bringart M., Turpin C., Rondeau P., Planesse C., Ait-Arsa I., Gimié F., Marodon C., Meilhac O., Gonthier M.-P. (2021). Caffeic Acid, One of the Major Phenolic Acids of the Medicinal Plant Antirhea borbonica, Reduces Renal Tubulointerstitial Fibrosis. Biomedicines.

[61] Zhu L., Li J., Wu D., Li B., Dayang W., Bing L. (2018). The protective effect of beta-casomorphin-7 via promoting Foxo1 activity and nuclear translocation in human lens epithelial cells. Cutan. Ocul. Toxicol..

[62] Sakaguchi M., Fujimori T., Satoh T., Matsumura E. (2001). Effects of β-casomorphins on neuronal survival in culture of embryonic chick dorsal root ganglion neurons. Jpn. J. Pharmacol..

