# Supplementary material for: Keratin Scaffolds Containing Casomorphin Stimulate Macrophage Infiltration and Accelerate Full-Thickness Cutaneous Wound Healing in Diabetic Mice
Source: Molecules. 2021 Apr 27;26(9):2554. doi: 10.3390/molecules26092554 (PMC8125279; doi:10.3390/molecules26092554)
Supplement: Supplementary file 1 [file molecules-26-02554-s001.zip › molecules-1164498-supplementary.pdf]

Keratin scaffolds containing casomorphin stimulate macrophage infiltration and accelerate full-thickness cutaneous wound healing in diabetic mice

Marek Konop[1,2,3*], Anna K. Laskowska[4], Mateusz Rybka[1], Ewa Kłodzińska[5,], Dorota Sulejczak[6], Robert A. Schwartz[7], Joanna Czuwara[3]

[1]*Department of Experimental Physiology and Pathophysiology, Laboratory of Centre for Preclinical Research (CePT), Medical University of Warsaw, Warsaw, Poland*

[2]*Department of Neuropeptides, Mossakowski Medical Research Centre, Polish Academy of Sciences, Warsaw, Poland*

[3]*Department of Dermatology, Medical University of Warsaw, Warsaw, Poland*

[4]*Department of Pharmaceutical Microbiology, Centre for Preclinical Research and Technology (CePT), Faculty of Pharmacy, Medical University of Warsaw, Poland*

[5]*Department of Analytical Chemistry and Instrumental Analysis, Institute of Sport - National Research Institute, Warsaw, Poland*

[6]*Department of Experimental Pharmacology, Mossakowski Medical Research Institute, Polish Academy of Sciences, Warsaw, Poland*

[7]*Department of Dermatology, Rutgers New Jersey Medical School, Newark, United States of America*

1. Materials and methods

Reagents

Sodium hydroxide, ethanol, and hydrochloric acid were from Avantor Performance Materials Poland S.A. (Gliwice, Poland). Pepsin was from BTL Sp. z o.o. (Łódź, Poland). Trisodium citrate, sucrose, eosin Y, hematoxylin solution (Harris-modified), xylene, phosphate-buffered saline (PBS) tablets and Hoechst 33258 bisbenzimide were from Sigma-Aldrich. Permount mounting medium was from Fisher Scientific (Pittsburgh, PA, USA) and Vectashield. Morphine hydrochloride was purchased from Polfa Kutno (Kutno, Poland). β-Casomorphin was from Bachem (Bubendorf, Switzerland).

Preparation of experimental wound dressings

Fur keratin derived powder (FKDP) was coated with 0.1% solution of casomorphin by soaking in the freshly prepared suspension (5 g of FKDP/ 10 ml of 0.1% casomorphin) for 24 hours with continuous moderate mixing. After the scheduled time, the mixing was stopped to allow the Caso-coated FKDP to sediment. The obtained product was lyophilized and powdered again as described previously (Konop *et al.*, 2020) and the final product was labeled as FKDP+0.1%Caso and was used as a wound dressing.

Electrophoretic measurements

Capillary electrophoresis analysis was performed using Agilent 7100 series CE instrument (Agilent Technologies, Santa Clara, CA, USA) equipped with a UV-VIS diode array detector and ChemStation software (AgilentOpenLABCDSChemStation version 1.7) for instrument control and data collection. A 50 µm i.d. fused silica capillary was purchased from Composite Metal Services (Worcester, Great Britain). The capillary was prepared as follows: a 64.5 cm long piece was cut and a ca. 2–3 mm of polyimide coating was burned out at both ends. A detection window was created at 8.5 cm distance from the outlet end, also by gentle burning out several millimeters of the coating, and wiped with a tissue wet with isopropanol. Then, the capillary, placed in the cassette, was put into the instrument and pretreated in the following way: 10 min of flushing with 0.1 M NaOH, then left for 5 min, rinsed with water for 5 min, and flushed with a running buffer for 5 min. Before each run was started, the capillary was flushed and refilled with a fresh buffer to remove any residual. The running buffer was 20 mM sodium tetraborate pH = 9.4. The sample was injected hydrodynamically 50 mbar x 20 s. The separations were performed at the voltage of 20 kV and the temperature of 20°C. During the CE experiments, the detection wavelength was at 214 nm.

Cell lines

Murine fibroblast cell line NIH/3T3 (ATCC CRL-1658) were cultured in DMEM supplemented with 10% (v/v) heat-inactivated, fetal bovine serum (FBS), 2 mM L-glutamine, 1 mM sodium pyruvate and 1% (v/v) antibiotic. Cells were maintained at 37°C. All necessary reagents for cell culture were bought from Sigma Aldrich (Warsaw, Poland)

Cell proliferation assay

Cell Proliferation Kit I (MTT- 3-(4,5-dimethylthiazol-2-yl)-2,5-diphenyltetrazolium bromide) (Cat. No. 11 465 007 001, Roche) was used to determine cell viability. Cells were

seeded in 96-well plates at a density of $3x10^3$ cells/well and incubated for 24 h. Next, opioids or opioid-incrusted FKPD were added to the cells (0-0.1%) and plates were incubated at 37°C. Cell viability was measured after 24 and 48 h. At each time point, 10 µl of MTT solution was added and incubated for 4 h at 37°C. Next, 100 µl of SDS solution was added to the plates and incubated overnight as per manufacturer instructions. Absorbance was measured at 570 and 690 nm using a microplate reader. Morphine and morphine incrusted FKPD served as an internal control. All experiments were performed in triplicate.

In vitro wound-healing assay

Cells were seeded in 24-well plates at a density of $2x10^4$ per well and incubated overnight at 37°C. Next, a "scratch" was created using a p200 pipette tip. The medium was carefully removed and a fresh medium, containing opioids or opioid coated dressings, was added. Plates were incubated for up to 48 h. Images were taken at times of 0, 24 h, and 48 h with a phase-contrast microscope (Nikon NIS-Elements F software). The rate of migration was calculated by measuring the distance between the edges of the wound at each time point.

Iatrogenically induced diabetes

Four hours before intraperitoneal injection of streptozotocin (STZ, Sigma-Aldrich) mice (N=20) were fasted and blood glucose level was measured with a glucometer (Accu-Chek, Roche). Body mass was measured. Diabetes was induced with 5 daily intraperitoneal (i.p.) injections of STZ (80 mg/kg body weight). Mice from the control group (N=20) were injected with the equivalent volume of citrate buffer (0.05 M, pH of 4.5) as described previously (Konop *et al.*, 2016). All mice were given a standard diet. Blood samples were taken from the lateral saphenous vein. The progression of diabetes was monitored by performing a blood glucose test using a glucometer on days 1, 4, 9, 16, 19, 23, 29, 30, and 31 of the study. A mouse was considered diabetic if three consecutive measurements showed a blood glucose level higher than 250 mg/dL.

Surgical procedure

Skin wounds were performed under general anesthesia initiated by halothane (5% v/v in oxygen-enriched (23.7% v/v) air and maintained at 3.5% v/v halothane in the oxygen-enriched air). Back skin fur was shaved with an electric clipper and the skin was disinfected with 70% ethanol. Two full-thickness skin flaps of 10 mm diameter each were removed with sterile scissors at the Th3-Th5 level, centering about 8 mm to the left and right from the midsagittal

plane. The wounds were fixed open by suturing their edges to the underlying fascia and muscles with silk sutures. The FKDP-Caso as powder dressing was applied once to one wound on the right side with care to fully and evenly cover the wound without masking its edges. The other wound on the left served as a control and remained undressed. The wounds were photographed with a camera located 10 cm above the skin level and pictures were used for the assessment of the initial wound area. After recovery from the anesthesia, mice were returned to the local animal facility and kept in the above-described conditions. Mice were housed separately in cages to avoid scratching and chewing one another. Animals did not receive any antibiotics or painkillers. The day of wound injury was marked as day “1”. Four, seven, and fourteen days after wounding were named respectively days 5, 8, and 15 of the study. Mice were anesthetized as described above, and their wounds were photographed for the measurement of the wound during the healing process. At each experimental time point, 6-8 mice were sacrificed by cervical dislocation on each of these time points and the skin biopsy encompassing the entire wound with a 1-2 mm margin was harvested for histopathological examination.

Histopathological analysis

Skin samples were fixed overnight in 4% formaldehyde solution in PBS. Skin biopsy was then cryoprotected by sequential soaking in a series of sucrose solutions in PBS (10, 20, and 30%, w/v) at 4°C until sinking. The cryoprotected material was frozen on dry ice, and cut into transverse 5-µm thick sections using the CM 1850 UV cryostat (Microm HM550, Thermo Scientific, USA). Half of the sections were used for routine hematoxylin-eosin (H&E) staining and examined for cell and tissue morphology using an Eclipse Ni-U Nikon light microscope (Japan) equipped with a charge-coupled device (CCD) camera and a PC-based image analyzer system.

To determine the collagen deposition, a Masson-Trichrome staining kit (Martinengo Italy) was used. Cryoprotected skin samples were stained according to the standard protocol by DiaPath. The sections were examined for collagen deposition using an Eclipse Ni-U Nikon (Japan) light microscope equipped with a CCD camera and a PC-based image analyzer system (CellSens Entry 1.6, Olympus).

To determine blood extravasation (microhemorrhages), six H&E-stained sections from each mice from the control and FKDP-0.1%Caso wounds for each time point were used. Only sections containing the full extent of the wound were chosen. The mean number of microhemorrhages for each wound was calculated for statistical analysis.

Immunohistochemistry staining

Glass-mounted skin sections were preincubated with 3% normal goat serum solution in PBS supplemented with 0.2% Triton X-100 (PBST). In the next step, the sections were incubated for 1 h at 37°C with PBST containing 1% normal goat serum and the following primary antibodies (Abs): murine monoclonal Ab against macrophages (Santa Cruz, USA, dil. 1:400); rabbit polyclonal Ab against p53 (Santa Cruz, USA, dil. 1:400); murine monoclonal Ab against NF-κB p65 (Santa Cruz, USA, dil. 1:400); rabbit polyclonal anti-collagen antibody IV (Santa Cruz, USA, dil. 1:100), and rabbit polyclonal Ab against NF-κB p50 (Santa Cruz, USA, dil. 1:400). Next, the sections were washed with PBST and subsequently were incubated for 1 h at 37°C with the appropriate secondary Abs: goat anti-mouse antibody conjugated with Alexa Fluor 594 (Invitrogen, USA, dil. 1:100) and goat anti-rabbit antibody conjugated with Alexa Fluor 488 (Invitrogen, USA, dil. 1:100). The stain was then drained off and coverslipped for fluorescence microscopy (Vector Labs Inc., Burlingame, CA, USA) for visualization.

The specificity of the immunolabelling was verified by performing a "blank" staining procedure with primary antibodies omitted in the incubation mixture. In these control sections, any staining was observed.

Immunostaining was detected with a model Optiphot-2 Nikon fluorescent microscope (Japan) equipped with the appropriate filters and recorded with a model DS-L1 Nikon camera (Japan).

RESULTS

Monitoring of the release of casomorphin from examined dressing

Electropherograms obtained for FKDP (basic dressing) and FKDP-0.1% Caso soaked in PBS are shown in Fig. 1S. To determine the release of the casomorphin from wound dressings, a standard curve method was used. Under optimized conditions, a calibration curve of casomorphin was obtained on an average of at least three replicate measurements. The linear regression equation was expressed y=1043.3x+5.4949 (R^2=0.9994).

The FKDP dressing (base dressing) does not give any signals on the electropherogram (black line on Fig. 1S). In the case of FKDP+0.1%Caso dressing, a single signal on the electropherogram was observed. It should be noted that the intensity of the signal decreased with the release of casomorphine from the dressing.

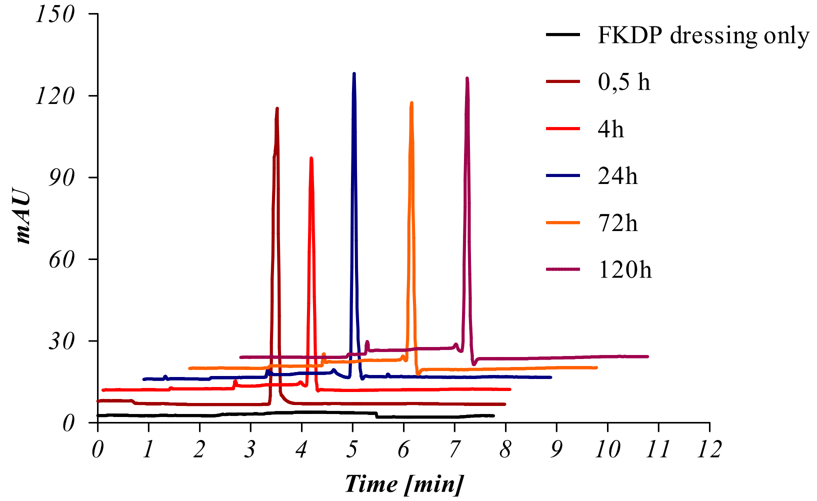

Figure 1S. CE electropherogram obtained for casomorphin released from keratin dressing (color online, black & white in print).

The effect of opioids on cell viability

Cells treated with casomorphin and morphine showed no significant differences in cell viability when compared to the control. The effect of morphine was not concentration- or time-dependent. There was no significant difference in the level of cell viability between incubation times or concentrations. For casomorphin, a significant difference in cell viability was only observed between incubation time at 100 µM (Fig. 2S)

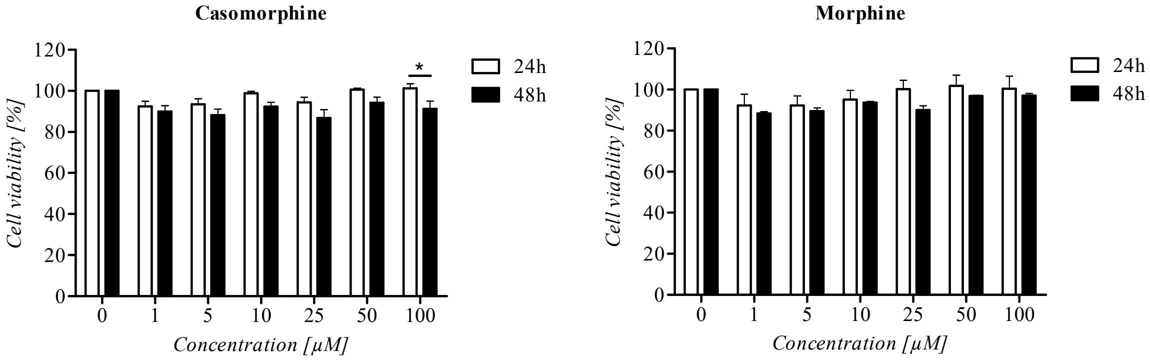

Figure 2S. Effect of opioids on cell viability after 24 and 48h. (The data are statistically significant when p< 0.05, two-way analysis of variance, followed by Bonferroni post hoc tests, mean ± standard error of the mean).

The effect of opioids on cell motility

The effect of opioids on cell motility was evaluated. The effect of opioids on cells was time-dependent ($p \leq 0.01$). It was observed that the rate of cell migration for both compounds was similar. After 24 h, cells treated with 10µM morphine migrated significantly faster than cells treated with lower concentrations of the compound. Cells exposed to casomorphin migrated at a similar rate. The migration rate was not concentration-dependent (Fig. 3S).

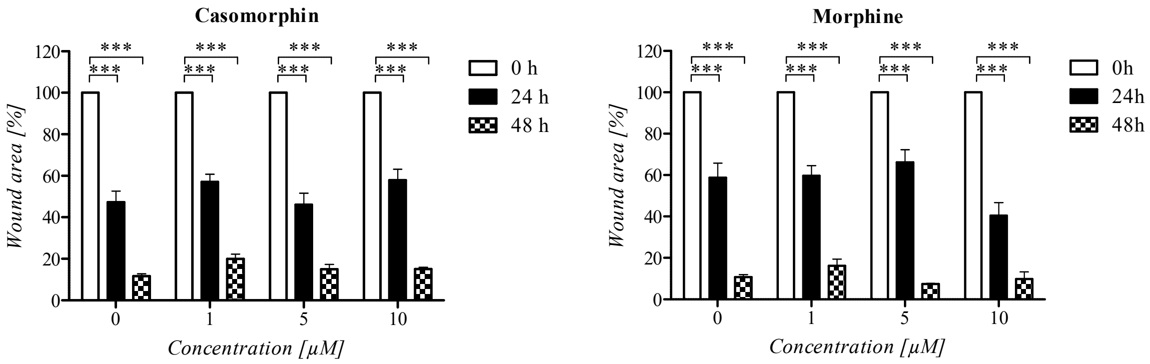

Figure 3S. Effect of opioids on cell migration after 24 and 48h. (The data are statistically significant when $p < 0.05$, two-way analysis of variance, followed by Bonferroni post hoc tests, mean ± standard error of the mean).

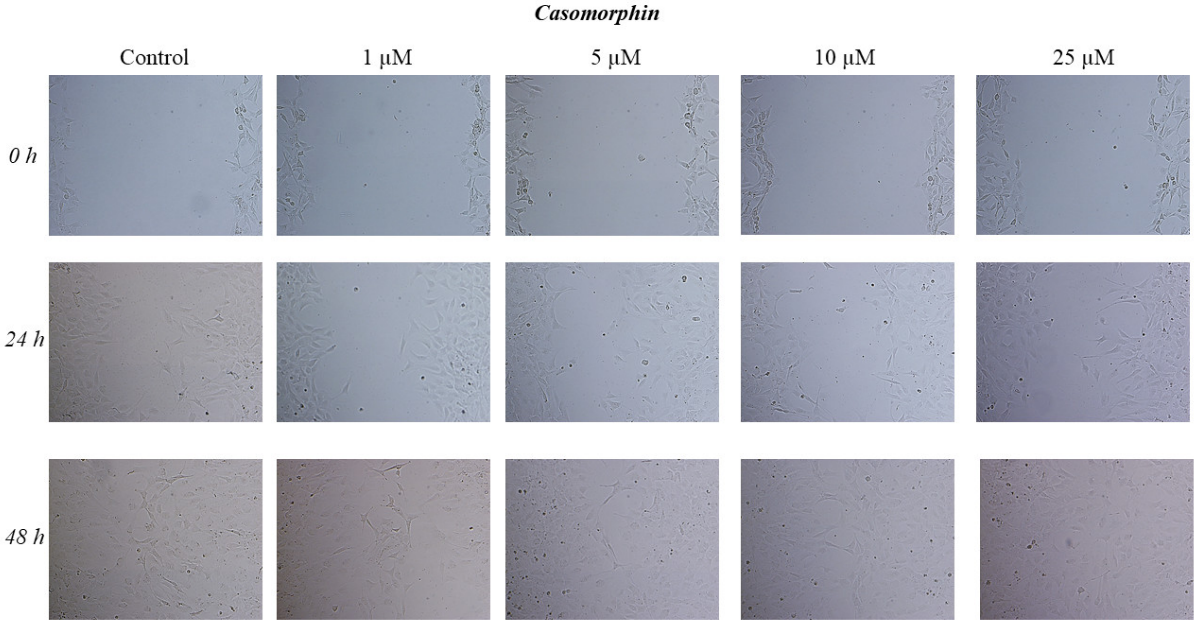

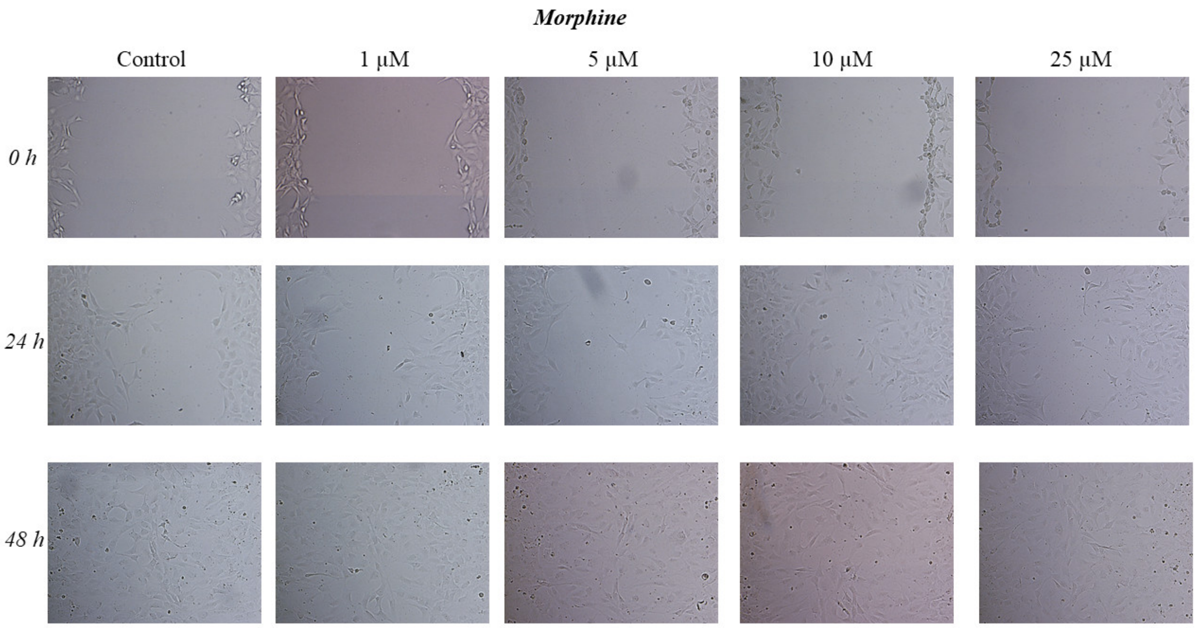

Figure 4S. The rate of wound closure in NIH/3T3 monolayer treated with opioids.

We also examined FKDP wound dressing and FKDP+0.1%Caso in vitro. It was observed that "wound" treated with keratin dressing (alone or with the addition of casomorphin) faster-undergone healing compared with control (Fig. 5S).

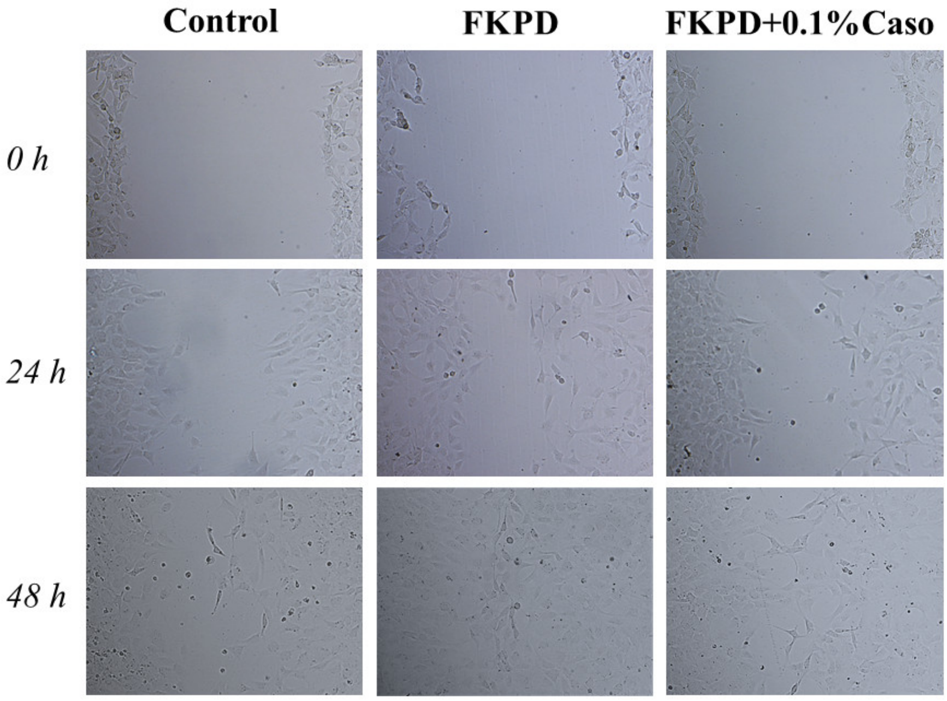

Figure 5S. The rate of wound closure in NIH/3T3 monolayer treated with experimental dressings.

References:

Konop, M. *et al.* (2016) 'The role of allogenic keratin - derived dressing in wound healing in a mouse model.', *Wound repair and regeneration : official publication of the Wound Healing Society [and] the European Tissue Repair Society*. doi: 10.1111/wrr.12500.

Konop, M. *et al.* (2020) 'Evaluation of keratin biomaterial containing silver nanoparticles as a potential wound dressing in full-thickness skin wound model in diabetic mice', *Journal of Tissue Engineering and Regenerative Medicine*. John Wiley & Sons, Ltd, 14(n/a), pp. 334–346. doi: 10.1002/term.2998.
